# FSH for the Treatment of Male Infertility

**DOI:** 10.3390/ijms21072270

**Published:** 2020-03-25

**Authors:** Livio Casarini, Pascale Crépieux, Eric Reiter, Clara Lazzaretti, Elia Paradiso, Vincenzo Rochira, Giulia Brigante, Daniele Santi, Manuela Simoni

**Affiliations:** 1 Unit of Endocrinology, Department of Biomedical, Metabolic and Neural Sciences, University of Modena and Reggio Emilia, Via P. Giardini 1355, 41126 Modena, Italy; clara.lazzaretti@unimore.it (C.L.); elia.paradiso@unimore.it (E.P.); vincenzo.rochira@unimore.it (V.R.); giulia.brigante@unimore.it (G.B.); daniele.santi@unimore.it (D.S.); manuela.simoni@unimore.it (M.S.); 2 Center for Genomic Research, University of Modena and Reggio Emilia, Via G. Campi 287, 41125 Modena, Italy; 3 Physiologie de la Reproduction et des Comportements (PRC), Institut National de Recherche pour l’Agriculture, l’Alimentation et l’Environnement (INRAE), Centre National de la Recherche Scientifique (CNRS), Institut Français du Cheval et de l’Equitation (IFCE), Université de Tours, 37380 Nouzilly, France; pascale.crepieux@inrae.fr (P.C.); eric.reiter@inrae.fr (E.R.); 4 International PhD School in Clinical and Experimental Medicine (CEM), University of Modena and Reggio Emilia, Via G. Campi 287, 41125 Modena, Italy; 5 Unit of Endocrinology, Department of Medical Specialties, Azienda Ospedaliero-Universitaria, Via P. Giardini 1355, 41126 Modena, Italy

**Keywords:** male infertility, FSH, testis, biosimilars, gonadotropin

## Abstract

Follicle-stimulating hormone (FSH) supports spermatogenesis acting via its receptor (FSHR), which activates trophic effects in gonadal Sertoli cells. These pathways are targeted by hormonal drugs used for clinical treatment of infertile men, mainly belonging to sub-groups defined as hypogonadotropic hypogonadism or idiopathic infertility. While, in the first case, fertility may be efficiently restored by specific treatments, such as pulsatile gonadotropin releasing hormone (GnRH) or choriogonadotropin (hCG) alone or in combination with FSH, less is known about the efficacy of FSH in supporting the treatment of male idiopathic infertility. This review focuses on the role of FSH in the clinical approach to male reproduction, addressing the state-of-the-art from the little data available and discussing the pharmacological evidence. New compounds, such as allosteric ligands, dually active, chimeric gonadotropins and immunoglobulins, may represent interesting avenues for future personalized, pharmacological approaches to male infertility.

## 1. Introduction

Follicle-stimulating hormone (FSH) is a dimeric glycoprotein released by the pituitary and targeting gonadal cells of both males and females. The molecule is structurally similar to luteinizing hormone (LH) which, together with FSH, regulates reproduction acting through specific G protein-coupled receptors (GPCRs) and modulating steroidogenesis, cell metabolism and growth [1]. Despite similarities between these two hormone-receptor systems, gonadotropins mediate sex-specific effects mainly due to a physiologically different expression of steroidogenic enzymes and receptors, according to different cell types. In particular, in the steroidogenic ovarian granulosa cell, the FSH receptor (FSHR) is co-expressed with the LH (and choriogonadotropin, hCG) receptor (LHCGR) during the fertile age, while only the FSHR is expressed in the non-steroidogenic Sertoli cells, in the testes. Therefore, the nature of FSH action is different in the two genders, hence the clinical approaches to infertility [2]. To this purpose, a number of strategies and hormonal drugs were developed and used for the treatment of infertile women, while more efforts are necessary for optimizing therapy for male infertility [3]. In this review, we discuss the action of FSH, as well as its current use and perspectives for treating male infertility.

## 2. FSH-Induced Signaling Network

FSH binding to its cognate receptor, the FSHR, leads to the rewiring of a complex intracellular signaling network that profoundly alters gene regulation at several levels. Hormone binding to the Leucine-rich repeat domain provokes conformational changes in the receptor, including the interaction of a sulfated tyrosine within the receptor hinge region with the interface of FSH α and β chains [4,5], and tethering of a decapeptide that protrudes out of the plasma membrane from the first intracellular transmembrane helix [6]. Work is still ongoing to decipher the crystal structure of the entire FSHR, including transmembrane domains, in active conformation. It is likely that these changes propagate to the transmembrane helices, leading to Gαs protein activation, in agreement with the activation mechanisms depicted for other Gαs-coupled GPCRs such as the β2-adrenergic receptor [7,8,9]. The FSHR is also rapidly desensitized upon phosphorylation by GPCR kinases (GRKs), that create anchoring sites for β-arrestins [10,11,12,13,14,15,16]. These scaffolding proteins initiate internalization of desensitized GPCRs by binding to clathrin-coated pit components such as the adaptor protein complex (AP2) [17].

A prominent effector of Gαs protein-coupled FSHR is adenylate cyclase, that catalyzes the cyclization of adenosine monophosphate (AMP) to cyclic AMP (cAMP), and ultimately regulates the activity of its intracellular targets, mainly protein kinase A (PKA) and, presumably, exchange factors directly activated by cAMP (EPAC). However, the FSH signal is transduced via proteins other than the canonical Gαs, as demonstrated initially by adenovirus-mediated expression of a constitutively active form of Gαs protein, GαsQ227L, in granulosa cells [18]. In this condition, ligand-independent cAMP production enhanced progesterone secretion to a level comparable to FSH, but estradiol production was decreased. In fact, these data are indicative of the link between the Gαs protein/cAMP/PKA-pathway to steroidogenesis, the latter being absent in Sertoli cells. Studies in primates revealed that FSH would act synergistically with testosterone (T) in priming the Sertoli cell response to endogenous stimuli that sustain spermatogenesis [19,20], indicating that the FSH-induced cAMP/PKA-pathway exerts a trophic [21], rather than steroidogenic function, which is committed to LH [22]. Moreover, pleiotropic coupling of the FSHR to other G proteins has been demonstrated [23]: for example, coupling of the FSHR to the Gαi protein is critically involved in Sertoli cell proliferation in the neonate rat [24]. In addition, LH-induced coupling of the LHCGR to Gαq is rearranged when the FSHR is co-expressed in the same cell, as occurs during follicular growth. However, the cross-talk between FSHR- and LHCGR-mediated signal transduction pathways in the same cell is absent in males, where these receptors are specifically expressed in Sertoli and Leydig cells, respectively, located apart from each other.

Beside their ability to promote desensitization and internalization, β-arrestins also assemble a plethora of signaling modules, as shown for many different GPCRs [25,26]. So far, however, upon FSHR stimulation, only the extracellular-regulated (ERK) mitogen-activated protein (MAP) kinase- and ribosomal protein S6 kinase beta-1 (p70S6K)-dependent signaling has been demonstrated to be partly mediated by β-arrestins. Of note, when comparing the mode of activation of both of these pathways in HEK293 cells, ERKs are activated sequentially through Gαs and β-arrestins [16], whereas the p70S6K pathway is activated concomitantly by these two transducing mechanisms [27]. This observation emphasizes the role of GPCR transducers to coordinate the dynamics of the downstream signaling network and has potential implications regarding the action of FSHR biased ligands described in the next sub-sections of this review.

Both of these signaling pathways exhibit developmental regulation [24,28]. For example, ERK MAP kinases mediate the mitotic function of FSH in neonatal Sertoli cells [24] and in immature granulosa cells [29], by increasing the expression of D1 and D2 cyclins, respectively [24,30]. In both FSH natural target cells, ERK activation is at least cAMP-dependent, and it is not known whether β-arrestins intervene. Interestingly, β-arrestins appear to be involved in cell survival, that is accompanied by ERK phosphorylation, as observed in the immortalized human granulosa cell line hGL5 [31].

As for the p70S6K pathway, the role of β-arrestins in its activation in seminiferous tubules or in follicles has not been clarified yet, although in Sertoli cells, β-arrestin 1 and p70S6K can be identified in common protein complexes [27]. p70S6K activation relies on protein kinase B (Akt) [28,32], and activation of this pathway is presumably responsible for enhancing mRNA translation measured in response to FSH [33,34], as a hallmark of FSH anabolic function and trophic role. Interestingly, FSH-activated Akt appears to protect cells from autophagy, by inhibiting the transcription factor forkhead box protein O1 (FOXO1) and promoting its nuclear exclusion. By these means, FOXO1 can no longer stimulate the expression of autophagy-related genes [35]. FOXO1 is key to the differentiating action of FSH, at least in granulosa cells and, quite unexpectedly, a recent genome-wide analysis revealed that most FSH-responsive genes had FOXO1-binding sites in their promoter (60% of identified genes), and not CREB-binding elements (CRE), as anticipated [36]. While FOXO1 is constitutively nuclear and bound to DNA, where, for example, it inhibits the expression of proliferation genes such as Cyclin D2 [37], these data suggest that the role of FSH would be to counteract the general action of this transcription factor. This echoes the fact that Akt likely antagonizes the pro-apoptotic role of cAMP, that otherwise has no effect on FOXO1.

Ultimately, the FSH-regulated signaling network alters the gene expression pattern of gonadal cells. The influence of FSH-induced signaling on gene expression, however, extends far beyond the regulation of mRNA transcription and translation, and microRNAs (miRNAs) now appear as key players in the control of reproductive function. Accordingly, Dicer, a key enzyme of miRNA processing, is involved in the expression of genes essential for meiosis and spermiogenesis, as demonstrated by Sertoli cell-selective knock-out in mouse of the Dicer gene [38]. By similar experiments, the importance of Dicer in ovulation has also been stressed in vivo [39,40,41], and the expression of several miRNAs leading to key regulators of follicular growth is sensitive to FSH. For example, FSH inhibits the expression of the miR10 family, otherwise involved in a pro-apoptotic negative feedback loop of transforming growth factor beta (TGFβ) activity [42]. In the male rat where FSH and testosterone action was suppressed, the miRNA network was identified at spermiation, a spermatogenesis stage particularly sensitive to hormone regulation. At this stage, elongated spermatids are released from Sertoli cells, a process accompanied by complex membrane rearrangements. Four miRNAs of this microarray analysis appeared complementary to the phosphatase and tensin homolog (*PTEN*) mRNA, that were localized in the apical region of Sertoli cells, in the vicinity of mature spermatids. The hormonal input would lead to the degradation or synthesis inhibition of these miRNAs, then stabilizing PTEN at spermiogenesis [43]. Interestingly, the PTEN protein level is increased following FSH cell stimulation in vitro, leading Sertoli cells to achieve terminal differentiation [44]. Since FSH enhances PTEN protein level within minutes, the mechanisms involved probably occur post-transcriptionally, similarly to the hormone-induced degradation of miRNA that prevents the accumulation of PTEN locally in Sertoli cells at spermiation. Hence, miRNA networks might regulate the compartmentalization of FSH signaling components in Sertoli cells and control the kinetics of these biochemical reactions. These examples point to a widespread role of a miRNA network that is counterbalanced by FSH function in both granulosa and Sertoli cells.

**Figure 1:**
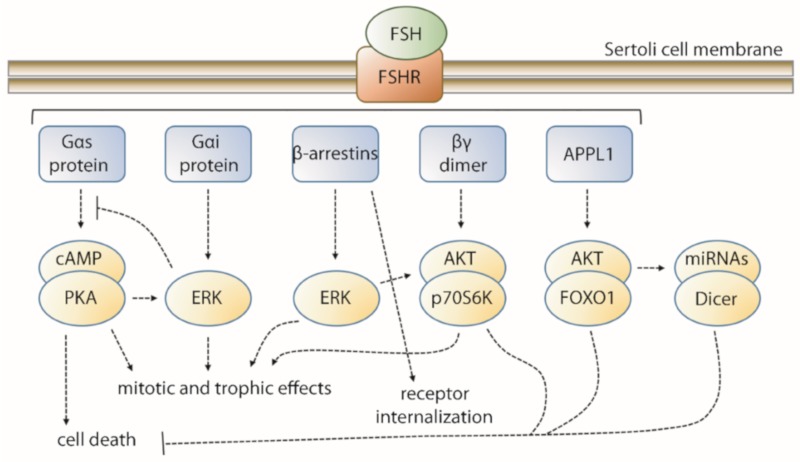
Main follicle-stimulating hormone (FSH)-mediated signaling network in Sertoli cells. Intracellular FSH receptor (FSHR) interactors are indicated in blue and associated with the downstream signaling modules. Most of the signaling cascades culminate with mitotic and trophic effects in Sertoli cells, while other signals are addressed to anti-apoptotic effects and β-arrestin-mediated FSHR internalization.

In summary, FSH mediates the activation of a complex network of signaling pathways (Figure 1), in gonadal target cells, that is not completely deciphered yet. In the context of female fertility, several studies attempted to determine congenital and environmental issues modulating the response to FSH [45,46], aiming to improve assisted reproductive technologies (ART) outcomes [47]. In the male, deficiency of FSH signaling may impair fertility and requires clinical treatments for restoring the gonadal function [48]. For these purposes, a number of FSH molecules were developed to modulate the target cell response to the hormone and used for infertility treatments.

## 3. FSH in Therapy

The employment of FSH for infertility treatments started in the early 1960s, when the first gonadotropic compound was extracted from human pituitary glands. For a long time after that, urinary-derived and highly-purified hormones have been used, in spite of supply problems and the risk of diseases due to potential contamination with prions [49], until the advent of recombinant FSH [50]. Currently, several recombinant and biosimilar FSH molecules are marketed, along with highly purified urinary FSH preparations. Biosimilars were developed after the patent of follitropin alpha and beta expired, and have a similar, albeit not identical molecular structure, and the same biological action as the originator [51]. The therapeutic employment of all these FSH preparations is mainly in the context of female infertility and assisted reproduction, while its use in the male is limited to hypogonadotropic hypogonadism [3]. FSH therapy in male idiopathic infertility is currently discussed [3] but no convincing evidence of its efficacy exists.

Human FSH is composed by an α subunit, common to all glycoprotein hormones, and a hormone-specific β subunit, which both undergo important post-translational changes. In fact, FSH is secreted by the pituitary as a mixture of different glycosylated variants [51,52,53,54], which differ in the number and the composition of oligosaccharides attached to the protein, resulting in changes in the molecule half-life and isoelectric point [55]. Two of the four N-glycosylation sites are located on the α chain at positions 52 and 78 (Asn^52^ and Asn^78^), whereas the two others, linked to the asparagine at positions seven and 24 are located on the β subunit [53,56]. Four main FSHβ-specific glycoforms exist: a fully glycosylated form, indicated as hFSH^24^, the forms hFSH^21^ and hFSH^18^, carrying glycosylations at the Asn^7^ and Asn^24^, respectively, and the hFSH^15^, lacking glycosylations on the β subunit [54]. The degree of sialylation and antennarity of the oligosaccharide chain can deeply influence the hormonal bioactivity [54,57,58]. Variations in serum levels of FSH glycoforms were found and suggest that they might have different physiological activity in men, where FSH isoform composition is demonstrated to change with age [51,59].

The rationale supporting the glycoform-specific activity relies on the assumption that different oligosaccharide chains may modulate the hormone-receptor structural interaction [58,60,61] and the downstream signaling cascades [57,58,62]. These considerations generated the idea that FSH variants could act as biased receptor agonists [63] and the impact of carbohydrate heterogeneity on FSH bioactivity was investigated both in vitro and in vivo [57,58]. For instance, a study showed that the hypo-glycosylated forms of FSH (hFSH^21^/hFSH^18^) exhibit a greater receptor binding activity compared to the fully glycosylated molecule in vitro [64]. However, in vivo studies have only partially confirmed what was found in vitro; female FSHβ-encoding gene (FSHB) null mice injected with different doses of hFSH^21^/hFSH^18^ or hFSH^24^ had higher estradiol production with less glycosylated forms. These two glycoforms also impact on specific subsets of FSH-responsive genes, but no different activation of PKA, CREB, p38 MAPK, ERK and AKT were found, as well as ovarian weight gain, between less and fully glycosylated FSH [65,66]. These experiments suggest that the biased activity of FSH variants observed in vitro is likely attenuated in vivo. Interestingly, different data emerged from male FSHB null mice, where hFSH^21^/hFSH^18^ promoted the increase of testicular weight more efficiently than the fully glycosylated FSH^24^, correlating with testis tubule size and the number of germ cells per tubule. In Sertoli cells of these animals, less glycosylated FSH promoted significantly higher expression levels of target genes and activation of proliferation markers than hFSH^24^ [65]. Taken together, these data are suggestive of a relevant physiological impact of FSH glycoforms, even though this impact is likely to be sex-specific.

During the last decades, the knowledge about FSH glycosylation was exploited for the development of new molecules for improving infertility treatments of both males and females [51,52,59]. Recombinant FSH is obtained from cultured mammalian cells and has a better safety, purity, specific activity, and batch-to-batch consistency than the urinary molecule [49]. Even if superiority of the recombinant hormone was claimed in the context of ovarian stimulation [67], the literature on this topic would require further, independent investigations and should be considered cautiously. In any case, isoform composition is different between recombinant and urinary FSH. The recombinant hormone contains a higher proportion of less-acidic isoforms, while human urinary FSH includes higher number of acidic forms. The less-acidic isoforms present a shorter circulatory half-life because they have a faster circulatory clearance than the acidic isoforms [68]. On the other hand, the acidic isoforms have relatively slow clearance, and induce better follicular maturation and estradiol secretion, than the less-acidic isoforms [69]. More acidic FSH isoforms have a prolonged half-life due to reduced kidney clearance and are secreted during the early and mid-follicular phase [69,70]. Furthermore, the half-life of the gonadotropin may even be prolonged by additional glycosylation sites obtained after amino acid chain manipulation [71]. Less is known about the efficacy of these molecules in the male. A comparison between recombinant and urinary FSHs was performed in men affected by congenital or acquired hypogonadotropic hypogonadism (HH) [72]. In this study, patients were treated for 3 to 6 months with hCG before the addition of daily FSH injection for 18 more months, resulting in beneficial effects evaluated as testis growth, spermatogenesis and fertility restoration. This study revealed no difference between the urinary and recombinant hormones [72].

To date, there are three marketed recombinant non-chimeric FSHs: follitropin alpha and follitropin beta, both expressed in the Chinese Hamster Ovary (CHO) cell line, and the recently developed follitropin delta expressed in the human fetal retinal cells PER.C6 [73]. These molecules have the same amino acid sequence but differ in glycosylation, composition of sialic acid residues and isoelectric coefficients [74]. Follitropin alpha is more acidic than follitropin beta, resulting in different biological activity, half-life and metabolic clearance [75]. Moreover, follitropin alpha has only alpha^2,3^-linked sialic acid, while follitropin delta includes a higher proportion of tri- and tetra-sialylated glycans, with both alpha^2,3^- and alpha^2,6^-linked sialic acid [76]. Another FSH molecule is under testing; it is called follitropin epsilon and is claimed to have optimized human glycosylation, with pharmacokinetics [77] and overall ovarian response similar to follitropin alpha [78].

A number of FSH biosimilars are under evaluation and have not yet passed the clinical phase III/preclinical testing (Table 1), while Gonapure^®^ (Minapharm Pharmaceuticals, Cairo, Egypt), DA-3801^®^ (Dong-A ST/Genexine, Seoul, South Korea) and Cinnal-F^®^ (CinnaGen, Tehran, Iran) were marketed recently and only in their countries of origin. Therefore, the number of independent studies comparing their performances to those of the originator are too low to be fully informative. Instead, the two follitropin alpha biosimilars marketed as Ovaleap^®^ (Theramex Ireland Limited, Dublin, Ireland) and Bemfola^®^ (Gedeon Richter, Budapest, Hungary) were compared to the reference product Gonal-f^®^ (Merck KGaA, Darmstadt, Germany), having different structures due to post-translational modifications [53,79]. In fact, in vitro data indicated that they would modulate a similar pattern of intracellular signaling activation and steroid synthesis, in FSHR-expressing cells, although the measurement of calcium ions (Ca^2+^) increased suggesting that these hormones may act as biased ligands [79]. Similar results were obtained by comparing follitropin alpha and follitropin delta in vitro, showing similar binding affinity and potency in the activation of FSHR-mediated cAMP increase and steroid synthesis, in transfected HEK293 and human primary granulosa cells, respectively [80]. However, in rodents, the injection of these FSH isoforms exhibited different pharmacokinetics, due to the clearance operated by different metabolic pathways, depending on the type of glycan linked to the hormones [80].

To overcome the relatively short half-life of FSH, which requires daily administration, a new recombinant molecule with a prolonged action was produced in transfected CHO cells [81], corifollitropin alpha (Elonva^®^; MSD, Readington, NJ, USA). This chimeric molecule includes the sequence encoding the CTP extension of hCGβ fused to the human FSHβ subunit, which bears four O-linked glycosylation sites and provides extended half-life [82,83]. Corifollitropin alpha has slower absorption and longer elimination half-time than follitropin alpha [84] so that one single injection can replace seven daily injections of follitropin alpha [85,86,87]. In fact, in cultured primary human granulosa cells, corifollitropin was demonstrated to be more potent than FSH in increasing aromatase gene expression [88], suggesting a high capability of inducing estrogen synthesis. This finding supports in vivo observations reporting that women treated with corifollitropin alpha have a higher number of metaphase II oocytes at ovum pick-up and of formed embryos, as well as higher overstimulation risk compared to FSH (parameters are highly dependent on the estrogenic functions but require further examinations in vivo) [89].

In summary, we can assume an overall clinical equivalence of various recombinant FSH molecules available. This equivalence is evident in vitro, especially when they are compared in cell systems expressing both FSHR and LHCGR, such as the human granulosa cells, and in vivo in clinical, non-inferiority studies. While, in the first case, FSH-induced signals may be modulated through the molecular cooperation of the two receptors [90,91] that attenuates the glycoform-specific activation of downstream signaling cascades [92], data obtained in vivo may be affected by the complex network of hormone actions and by the genetic background of the host, impeding a proper dissection of the hormone-specific effects. Most of the results demonstrating specific biological effects of FSH iso/glycoforms were obtained in cell models expressing only the FSHR and lacking LHCGR expression [64,79]. Therefore, these data are suggestive of hormone-specific activity in Sertoli cells, since expressing only the FSHR indicates a potential way for personalizing treatments of infertile male patients.

## 4. FSH Use in Hypogonadotropic Hypogonadism: State-Of-The-Art

Male infertility is a multifactorial clinical condition affecting at least half of infertile couples. Within the long list of known pathogenic causes of male infertility, HH may represent at least 10% of cases in highly specialized centers [93,94,95]. In this disease, a congenital or acquired defect of hypothalamic gonadotropin releasing hormone (GnRH) production/function and/or pituitary gonadotropin secretion is observed, leading to impaired testicular functions [93,94,95]. On the other hand, 30–40% of male infertility cases remain idiopathic, without a detectable pathogenesis characterized by abnormal semen parameters [96]. Whereas a specific and efficient treatment to restore fertility exists for HH patients, in male idiopathic infertility only empirical approaches are available.

In central HH, fertility can be restored using either GnRH or exogenous gonadotropins. Pulsatile GnRH administration represents, conceptually, the best replacement therapy, leading to the secretion of endogenous gonadotropins from the pituitary gland and the consequent stimulation of physiological testicular functioning, inducing intra-testicular testosterone production and Sertoli cell support [97,98,99,100]. However, this therapeutic approach is not routinely used, since it requires an external pump delivering GnRH pulses subcutaneously, with high costs and discomfort for the patient. On the other hand, the administration of exogenous gonadotropins may be a successful treatment in most cases. In particular, testosterone-mediated virilization and sperm production may be efficiently restored by hCG alone or administered together with FSH, although the optimal scheme, timing and dosage for this treatment remains controversial. Indeed, the issue is debated and was recently evaluated by a meta-analysis assessing the effects of hCG alone or in combination with FSH, administered in a variety of clinical settings, on sperm concentration of HH subjects [101]. Gonadotropin stimulation was beneficial in 75% of the cases, which obtained a final mean sperm concentration of 5.92 million/mL [101], sufficient to obtain a pregnancy, although sub-physiological [102]. These data suggest the existence of a beneficial, synergistic action of FSH and hCG-induced testosterone, restoring fertility despite sub-optimal sperm parameters [101]. However, clinical trials evaluating the effects of gonadotropins in inducing fertility in HH have some limitations. The therapy relies on the administration of exogenous gonadotropins, which does not replace the pulsatile pituitary secretion fundamental for delivering a physiological signal naturally regulating fertility in mammals [103], even if this approach seems to be as efficient as pulsatile GnRH therapy [101]. Most studies using gonadotropins were based on hCG, instead of the physiological hormone LH, which was present in the old hMG preparations but is absent from the modern, highly purified urinary gonadotropins. In light of the different intracellular signaling pattern mediated by LH and hCG [83] in Leydig cells [104,105], and considering that hCG has no functions in the adult male, where androgen production is supported by LH action, hCG might not be the optimal therapy and this possibility should be verified in future studies. In any case, intratesticular testosterone plays a central role in sustaining spermatogenesis, and this can be induced through the steroidogenic action of hCG, while FSH contribution would be mainly supportive of sperm quality [106,107].

## 5. FSH Use in Idiopathic Infertility: State-Of-The-Art

Many cases of male infertility remain idiopathic, revealing the current inability to define the underlying causes. In these cases, a rational pharmacological treatment does not exist, and clinicians attempt to stimulate spermatogenesis with various and not univocal results. Estrogen receptor modulators (SERM), such as tamoxifen and clomiphene citrate, are used off-label in some countries for treating male infertility [108,109]. These drugs block the estrogen receptor alpha, reducing the negative feedback at the hypothalamo-pituitary level and increasing the release of FSH and LH [110]. The efficacy of SERMs in treating idiopathic male infertility, evaluated by 11 randomized trials and combined in a recent meta-analysis, indicates that these drugs may be beneficial for increasing sperm concentration, sperm motility and pregnancy rate [111]. Also FSH administration was proposed as a treatment for idiopathic infertile men with impaired semen parameters and normal FSH serum levels [112,113]. The rationale of this application resides in the FSH effect on spermatogenesis, although its efficacy remains controversial and difficult to prove. Moreover, this approach has the limitation of relatively high costs and off-label usage [114], so that only limited clinical data are available [112,113]. To date, seventeen clinical trials evaluated FSH use in idiopathic infertile men and were listed in an opinion article [115], obtaining different results. These data were recently meta-analyzed and suggested that the use of FSH may increase overall pregnancy rates, which, however, was never considered as the primary end point in the original studies [116,117]. The evidence generated by clinical trials and meta-analyses is still weak, considering the very low number of patients treated by FSH, as well as the limitations intrinsic to considering pregnancy as the primary endpoint. This parameter is potentially biased by the female factor, limiting the comprehension of FSH benefits for male idiopathic infertility [116,117]. Moreover, since more than 15 idiopathic infertile men must be treated to achieve at least one pregnancy [116], a clinical study with large sample size is not easily feasible and data available to date are not sufficient to recommend an extensive FSH use. Despite the low and even diverging [118] experimental evidence, European clinical guidelines suggest the treatment with FSH as potentially beneficial for quantitative and qualitative sperm parameters and pregnancy rate, in selected men with idiopathic oligozoospermia or oligoasthenoteratozoospermia [112,113].

Clinical studies in idiopathic infertile men suggested sperm production dependency on the FSH dose [119,120], providing a rationale for optimizing the clinical approaches to the disease, which are currently mostly empirical. Indeed, data from the clinical studies indicated that the use of FSH would not be beneficial in about half of the patients. Clinical evaluations should be corrected for confounding factors, such as pharmacogenetic markers, which might lead to unclear results if ignored. These considerations emerged from studies in FSH-treated idiopathic infertile men, after consideration of the FSHR allelic state [106]. The FSHR carries a single nucleotide polymorphism (SNP), falling at position 680 of the receptor amino acid chain (p.N680S; rs6166), influencing testicular volume in men [121] and FSH-induced intracellular signaling in vitro [122]. Three months of daily FSH administrations improved the sperm quality of p.N680S N homozygous idiopathic infertile men, but not that of homozygous S [106], suggesting that the FSHR could be a pharmacogenetic marker. Other SNPs falling within genes regulating FSH-dependent signals are potential markers of sperm count and/or quality, as well as response to FSH treatment [123]. The FSHR promoter rs1394205 polymorphism is characterized by a G to A nucleotide change at position -29 upstream the start codon (-29G > A), that modulates the gene transcriptional activity [124] and is associated with FSH serum levels in men [125,126]. Another example is provided by the rs10835638 (-211G > T) polymorphism of the FSHβ-encoding gene (FSHB) [127] influencing serum hormone levels, sperm parameters and gonadal function in different male populations [128,129,130]. The combinations of p.N680S and other FSHR SNPs are linked to individual-specific pathophysiological effects [126,131,132], opening perspectives for a new pharmacological approach, optimizing the pharmacogenetic potential of FSH for infertility treatments.

## 6. Future Directions in the Control of FSHR Signaling

The development of novel FSHR modulators has been pursued for years by both academia and the pharmaceutical industry. These efforts can be categorized in three main groups: small non-peptide ligands, single-chain recombinant FSH and antibodies/antibody fragments. In the meantime, the concepts about GPCR activation and pharmacology have deeply evolved. Overwhelming structural, biophysical and pharmacological evidence now supports the co-existence of multiple active and inactive receptor conformations [133,134,135]. Moreover, compounds displayed different relative potencies in various assays, promoting the notion that pharmacological efficacy is multi-dimensional instead of bi-dimensional as predicted by the two-state model of receptor activation [136,137]. This led to the notion of pharmacological bias or ligand-directed signaling, in which different ligands stabilize distinct conformation ensembles, hence distinct transduction mechanisms [138,139,140]. Another important development has been the advent of allosteric modulators. These are ligands that bind a GPCR at sites distinct from the one used by the endogenous ligand and can also elicit biased responses. They can affect either positive (positive allosteric modulator, PAM) or negative (negative allosteric modulator, NAM) receptor activity. Moreover, they can act alone (ago-PAM) or in conjunction with the endogenous ligands (PAM or NAM) [141,142]. Importantly, some biased ligands have been reported to increase the window between side effects and therapeutic benefits, therefore opening great opportunities in drug discovery [143,144]. Of course, these advances and novel opportunities also apply to the targeting of FSHR.

Small non-peptide ligands have been explored in order to control FSHR activity with the ultimate objective of developing orally active drugs. Early studies demonstrated that some thiazolidinone derivatives displayed ago-PAM, NAM or potentially biased (i.e., preferential coupling to Gαs or Gαi) activities at the FSHR [145,146]. Several thiazolidinones demonstrated efficacy in rat granulosa cells and induced folliculogenesis in vivo in immature rats. However, further developments were hampered by unfavorable pharmacokinetics parameters [147]. Benzamides also showed selective PAM activity for FSHR compared to TSHR and LHCGR [148]. Dihydropyridine Org 24444-0 increased FSH-induced FSHR activation in vitro and induced follicle maturation in vivo [149]. Despite such promising effects on FSHR-induced signaling, neither benzamides nor dihydropyridines reached the market.

Non-peptide ligands were also reported to turn off FSHR signaling. Tetrahydroquinolines showed antagonistic effects on cAMP production without impairing FSH binding but no effect was observed in vivo [150]. ADX61623 showed decreased FSHR-induced cAMP production while improving FSH binding affinity. It was also able to decrease progesterone but not estradiol production in vitro [151]. Two other compounds from the same ADX series were later reported as biased NAMs at the FSHR: ADX68692 and ADX68693. ADX68692 decreased cAMP, progesterone and estradiol production in rat granulosa cells while ADX68693 reduced cAMP and progesterone but not estradiol production in the same model. Interestingly, ADX68692 efficiently reduced the number of oocytes recovered in mature female rats whereas the ADX68693 did not [152]. These two compounds also had biased NAM activities on LHCGR signaling [153] and suggest the opportunity to control the human reproduction with non-steroidal, pharmacological contraceptives.

**Figure 2:**
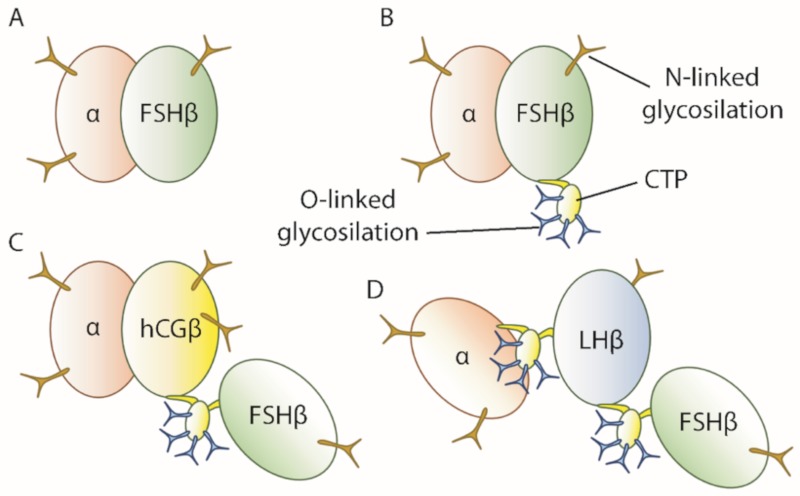
Schematic representation of FSH, FSH-CTP, and of the FSHβ-CTP-CGβ-α and FSHβ-CTP-LHβ-CTP-α analogues; (**A**) FSH molecule; (**B**) FSH-CTP with four additional O-linked glycosylations; (**C**) dually active FSHβ-CTP-CGβ-α analogue. (**D**) dually active FSHβ-CTP-LHβ-CTP-α analogue.

Another line of research has been centered on the development of single-chain gonadotropins [154]. Initial success obtained with single-chain recombinant hCG (hCGβ-subunit fused to the N-terminus of the gonadotropin α-subunit) led to the development of single-chain analogues of FSH [155]. In addition to the FSH-CTP, some analogues presenting dual FSH and LH activities were recently developed. Single chain FSH was engineered using the hCGβ CTP as a linker between FSH β-subunits and the α-subunit. These analogues had comparable or improved biological activity in vivo compared to native FSH, half-lives were also improved presumably because O-linked glycosylation sites of the CTP reduced hepatic clearance rates [156,157,158]. Some analogues (Figure 2) presenting dual FSH and LH activities (FSHβ-CTP-CGβ-α and FSHβ-CTP-LHβ-CTP-α) increased serum estradiol, ovarian weight and the formation of corpora lutea when injected in sheep [158,159,160]. To date, no single-chain FSH or dually active gonadotropin analogue has reached the clinical phase of development.

Antibodies and antibody fragments targeting GPCRs currently receive a lot of interest, culminating with two anti-GPCR therapeutic antibodies recently approved [161,162,163]. The idea that antibodies also represent a potential alternative to develop modulators of FSHR signaling is supported by the literature. In fact, antibodies raised against the β-subunit of bovine or ovine FSH potentiated the biological activity of FSH in mice [164,165]. Equine chorionic gonadotropin (eCG) shows natural interspecies promiscuity since it can bind to FSHR and LHCGR and modulate the signaling in non-equine species and for this reason, it has been widely used to control reproduction in farm animals [166,167,168]. Anti-eCG antibodies/eCG complexes were collected from the serum of goats treated with eCG and were shown to either stimulate or inhibit LH and FSH bioactivities depending on the animal treated [169]. Interestingly, anti-eCG/eCG complexes from three animals displayed stimulatory bioactivities. They were purified and in vitro characterized. A robust increase in ERK phosphorylation was measured when compared to eCG alone. Moreover, two of the complexes induced both PKA- and β-arrestin-dependent signaling, while the third complex stimulated only PKA-dependent signaling [170]. This demonstrates not only that anti-hormone antibodies can modulate FSH activity, but also that this modulation can be biased. Anti-FSHR antibodies and antibody fragments have been also used to control FSHR activity. A polyclonal antibody directed against the hinge-region of FSHR displayed agonistic activity, confirming the central role of the hinge region in the FSHR activation [171]. More recently, a library of synthetic nanobodies has been screened against whole cells expressing the FSHR, and anti-FSHR nanobodies were identified and behaved as non-competitive antagonists on FSHR-mediated cAMP production [172].

Interestingly, a fusion protein consisting of β-subunits fused to immunoglobulin Fc fragments was co-expressed with FSHα. Female rats injected with this FSH-Fc analogue displayed significantly increased ovarian weight compared to FSH-treated animals, suggesting that this analogue exhibits similar advantageous pharmacokinetic properties as immunoglobulins [173].

Two other classes of pharmacological modulators, pepducins [174] and aptamers [175] have recently shown promise as GPCR modulators. Even though, to our knowledge, they have not been assessed yet in the context of FSHR targeting, they may represent interesting avenues for future developments.

## 7. Future Clinical Perspectives

The fervor of basic research in studying new gonadotropin formulations and receptor modulators gives hope for the future. From a clinical point of view, it is necessary to improve therapy manageability and, before that, to understand the real efficacy of gonadotropins in male idiopathic infertility.

First of all, the proper use of FSH must be clarified. It is necessary to definitively determine whether the treatment with FSH improves reproductive parameters in males with serum FSH levels in the normal range. This must be done with studies on samples larger than those available so far, in order to define which therapeutic scheme is more effective. Further studies are needed to clarify optimal scheme, timing and dosage, in light of the FSHR and FSHB genetic background and developing patient-specific pharmacogenetic approaches.

An aspect still to be explored is the use of LH instead of hCG, in association or not with FSH. Since LH and hCG are different, and not fully interchangeable in their signal transduction properties, the use of LH in the treatment of male idiopathic infertility should be tested and could provide interesting results. Firstly, pharmacodynamics and the safety profile of LH in men must be assessed. Then, we should explore whether LH supplementation is able to improve spermatogenesis and pregnancy rates in infertile men over FSH alone or with FSH in association to hCG. The short half-life of LH should not be a hindrance in this case, since an increased systemic androgenization is not expected, but rather a trophic effect on Leydig cells and intratesticular testosterone is anticipated.

When the best possible scheme of association between FSH and hCG or LH is defined, it will then be necessary to produce more manageable formulations than those available today. Current therapeutic schemes are based on recurring intramuscular injections. Obviously, these modalities are invasive and need to be overcome. For this reason, new orally active drugs, e.g., small non-peptide ligands, must be studied and perfected.

New insights for achieving personalized non-hormonal approaches to male infertility may be provided by modulating the impact of immune cells on fertility. For instance, it was reported that leukocytes may entangle with sperms, reducing sperm motility and the chances of fertilization [176]. All these ideas must be aimed at finding the best treatment for all types of male infertility (potentially including the idiopathic cases) and personalizing the therapy from subject to subject, reaching the best results together with the least possible invasiveness.

## Figures and Tables

**Table 1:** FSH originator and biosimilars.

Drug Name	Status	Originator
GONAL-f	Marketed	Merck
Cinnal-f	Marketed	CinnaGen
DA-3801	Marketed	Dong-A Pharmaceutical
Bemfola	Marketed	Gedeon Richter
Ovaleap	Marketed	Theramex
Gonapure	Marketed	Minapharm Pharmaceuticals
Follitropin alpha biosimilar—Allergan/Itero Biopharmaceuticals	Phase III	Itero Biopharmaceuticals
LM-001	Preclinical	Alphamab
Follitropin alpha biosimilar—Cadila Healthcare	Phase III	Cadila Healthcare
Folitime	No development reported	GEMA
Primapur	Phase III	iVFarma
ProLease	Discontinued	Merck Serono

Data source: AdisInsight database—Springer. Available online at: https://adisinsight.springer.com (accessed on 1 March 2020).
